# Combination of preoperative neutrophil-lymphocyte ratio, platelet-lymphocyte ratio and monocyte-lymphocyte ratio: a superior prognostic factor of endometrial cancer

**DOI:** 10.1186/s12885-020-06953-8

**Published:** 2020-05-24

**Authors:** Rong Cong, Fanfei Kong, Jian Ma, Qing Li, Qijun Wu, Xiaoxin Ma

**Affiliations:** 1 Department of Obstetrics and Gynecology, Shengjing Hospital of China Medical University, Sanhao Street, 110004 Shenyang, People’s Republic of China; 2 Department of Clinical Epidemiology, Shengjing Hospital of China Medical University, Shenyang, People’s Republic of China

**Keywords:** Endometrial cancer, Monocyte-lymphocyte ratio, Neutrophil-lymphocyte ratio, Platelet-lymphocyte ratio, Prognosis, Nomogram

## Abstract

**Background:**

The preoperative peripheral blood neutrophil-lymphocyte ratio (NLR), platelet-lymphocyte ratio (PLR) and monocyte-lymphocyte ratio (MLR) have been reported to be associated with the prognosis of various cancers but are always discussed separately. The aim of this study is to bring the combination of NLR, PLR and MLR into the prognostic assessment system of endometrial cancer (EC) and establish a nomogram to provide an objective prediction model for clinical decisions.

**Methods:**

A total of 1111 patients with EC who had accepted surgical treatment during 2013–2017 were involved in the analysis. Their NLR, PLR, and MLR levels were obtained from a routine blood examination within 2 weeks before operation. Receiver operating characteristic curve (ROC) analysis was performed to determine optimal cutoffs. Chi-square tests analysed the associations of the ratios with other clinicopathological variables. The prognostic value was indicated by overall survival (OS) via Cox proportional hazards models and Kaplan-Meier analysis. R software was used to establish the nomogram based on the combination of NLR, PLR, MLR and other clinicopathological factors.

**Results:**

The median follow-up period was 40 months, and the median age was 56. The enrolled patients were stratified by cutoffs of 2.14 for NLR, 131.82 for PLR and 0.22 for MLR. Multivariate analyses demonstrated that high NLR over 2.14 (HR = 2.71, 95%CI = 1.83–4.02, P<0.001), high PLR over 131.82 (HR = 2.75, 95%CI = 1.90–3.97, P<0.001), and high MLR over 0.22 (HR = 1.72, 95%CI = 1.20–2.45, *P* = 0.003) were significantly associated with worse OS. The combined indicator, high NLR + high PLR + high MLR (HR = 4.34, 95%CI = 2.54–7.42, *P*<0.001), showed the highest prognostic value. The Harrell’s concordance index of the nomogram was 0.847 (95% CI = 0.804–0.890), showing good discrimination and calibration of this model.

**Conclusion:**

The combination of NLR, PLR, and MLR is a superior prognostic factor of EC. The nomogram involving the combination of NLR, PLR, MLR and other clinicopathological factors is recommended to predict OS for EC patients clinically.

## Background

Endometrial cancer (EC) is reported as the most common gynaecological tumour with increasing incidence in developed countries [1]. Despite intensive efforts in past decades to improve diagnostic criteria and surgery, radiotherapy and chemotherapy treatments, the management of EC faces the serious risk of recurrence, and cure can be difficult and challenging due to changes in the histological classification, controversy in the usage of lymphadenectomy for early stage patients, decline of adjuvant therapy and discrepant definition of recurrence risk factors based on various classifications [2]. Effective prognostic indicators for early detection of probable recurrence may be helpful for timely and optimal treatment and improve the survival rate. Clinically, noninvasive diagnosis of EC mainly relies on ultrasound and serum cancer antigen 125 (CA-125). However, CA-125 may be not a fitting prognostic indicator of EC because it rises in many physiological and pathological conditions, such as menstruation, ovarian cancer, pelvic inflammatory disease and pancreatitis [3]. In Kim’s [4] and Ding’s [5] studies, the combination of peripheral blood neutrophils and monocytes had higher prognostic value than CA-125.

A simple routine preoperative blood examination is of great importance for predicting the prognosis of patients with EC but is always ignored by clinicians. Since Virchow first noted leukocytes in neoplastic tissues and discussed the relationship between inflammation and cancers in 1881, the peripheral blood neutrophil-lymphocyte ratio (NLR), platelet-lymphocyte ratio (PLR), and monocyte-lymphocyte ratio (MLR) have been widely used to predict the prognosis of cancers, including gastric cancer, colorectal cancer, lung cancer, oesophageal cancer and breast cancer [6–10]. The indicators from peripheral blood examinations can be collected conveniently for dynamic evaluation of high-risk patients and relieve their financial burden.

However, there is a downside: most existing studies discuss the prognostic value of these ratios separately, and no study focuses on the combination of NLR, PLR, and MLR in EC. In Takahashi’s study [11], elevated NLR was not an independent factor of shorter survival for EC patients in multivariate analysis. In Aoyama’s study [12], elevated PLR was associated with PFS but not with OS and lymph node metastasis in multivariate analysis. A single ratio is not sufficient nor accurate to evaluate prognosis. Therefore, we integrated the combination of three ratios and some other clinicopathological factors to establish a nomogram model to provide an objective statistical evaluation scale for EC. The nomogram is usually used to provide an individualized evaluation of a certain event by integrating diverse determinant and prognostic variables, and it has been proposed as a better method or even a new standard compared to the traditional staging system in many cases, as reported in several studies [13, 14]. The aim of the present study is to evaluate the prognostic value of preoperative peripheral blood NLR, PLR, and MLR and their combinations in patients with EC and provide a reliable scoring nomogram model for doctors to make standardized clinical decisions.

## Methods

### Study population

We retrospectively collected clinicopathological data from patients with EC at our hospital from December 2013 to December 2017. Patients were eligible if they had been diagnosed with primary EC and undergone hysterectomy (with or without adnexectomy and lymphadenectomy). Involved patients were treated according to a detailed preoperative assessment including gynaecological examination, an imaging examination and preoperative histological findings. Patients without full blood count (FBC) data from a time frame of 2 weeks before surgery were excluded. Patients with incomplete clinicopathological data or follow-up information, with atypical hyperplasia or a carcinoid tumour, with other simultaneous malignancies or hematologic disorders, or those that received radiotherapy or chemotherapy before surgery were also excluded.

### Data collection

Clinical information was obtained from archived electronic medical records of the hospital information system. (i) Basic information included the age at surgery, the age at menopause, the body mass index (BMI), reproductive history, complications and smoking history. (ii) Pathological data included the clinical stage, tumour grade, histopathological subtype, depth of myometrial tumour invasion, lymphovascular space invasion (LVSI) and surgical method. (iii) Laboratory data included the FBCs (leukocyte, neutrophil, eosinophil, basophil, lymphocyte, monocyte and platelet counts, expressed in × 10^9^/L). Clinical stage and tumour grade were calculated according to the FIGO 2009 and the histologic typing system of the WHO. The end point index was overall survival (OS), which was defined as the time from the date of the primary surgery to the date of the last follow-up (June 30, 2018) or death from any cause. Death data were obtained from the death certificates.

### Statistical analysis

Statistical analysis was performed using IBM SPSS 19.0 (SPSS, Inc., Chicago, Illinois, USA) and R 3.0.1 software (http://www.Rproject.org). All included patients were stratified according to the preoperative NLR, PLR, and MLR cutoffs, which were generated by a receiver operating characteristic (ROC) curve. The maximum Youden index indicated the optimum cutoff. Survival analysis of the ratios was performed using Kaplan-Meier analysis, and the *P* value was used to identify significant differences between groups. The Cox proportional hazard models and the Schoenfeld residuals test were conducted to respectively evaluate the hazard ratios and proportional hazard assumptions of the variables. Associations between continuous and categorical variables were analysed using the Mann-Whitney U test and the chi-square test. To test the multicollinearity, we evaluated the variance inflation factors (VIF) and the standard errors. A predictor with VIF > 10 indicated serious collinearity. A multivariable analysis was conducted on the following covariables: age at surgery (< 55, 55–65, 65–75, ≥75), surgical stage (I-IV), tumour grade (1–3), BMI (< 25, 25–30, ≥30), diabetes (absent, present), lymphovascular invasion (absent, present) and histopathological subtype (endometrioid, stromal sarcoma, clear cell, serous, mixed, carcinosarcoma). All tests were two-sided, and a *P* value < 0.05 was considered statistically significant. R software was used to establish the nomogram to indicate the relationship between possible prognostic factors and actual OS, and Harrell’s concordance index (C-index) and a calibration curve were used to measure discriminative capacity. If the C-index is over 0.7 and the calibration curve is approximately matching with the basic curve, the nomogram will be of good prognostic significance.

## Results

**Fig. 1:**
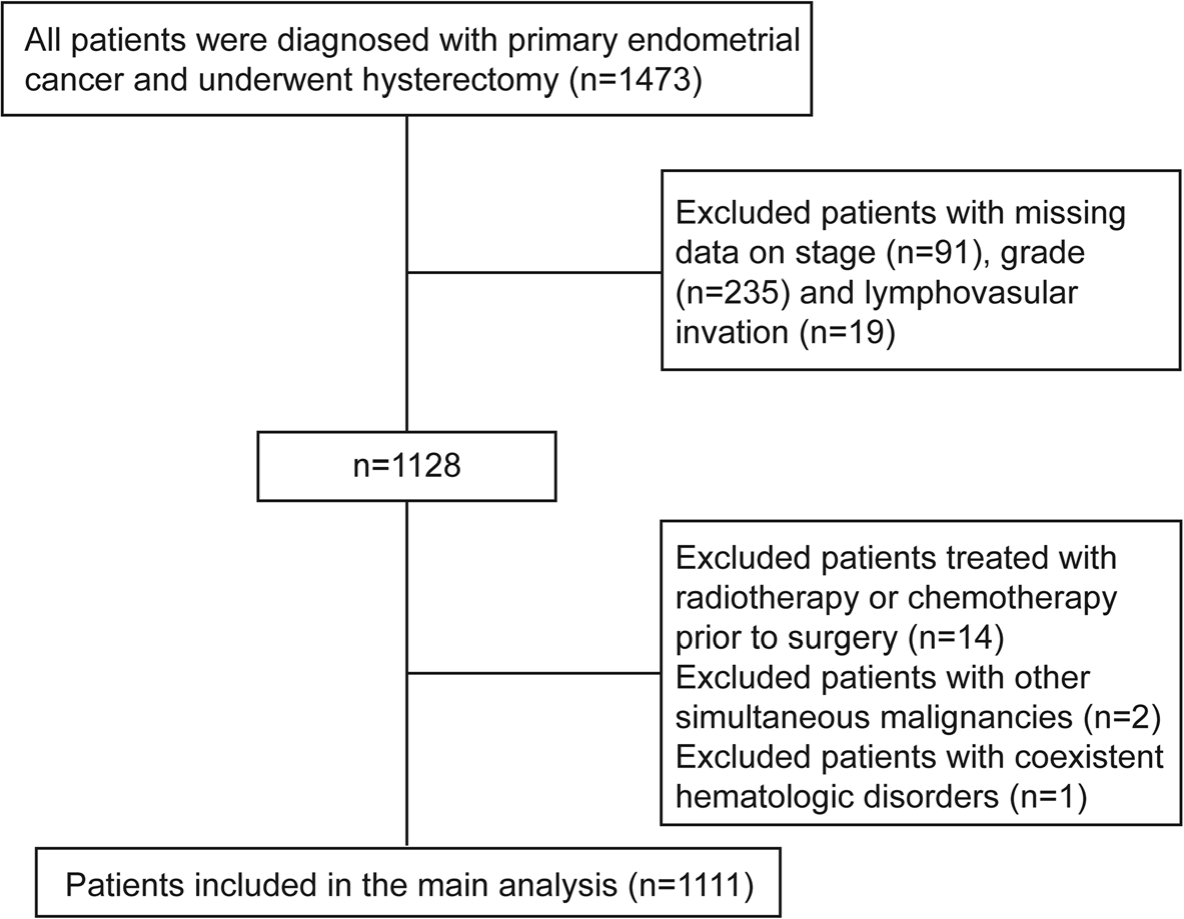
Flow chart of the included and excluded populations

In total, 1473 patients with primary EC who underwent hysterectomy between 2013 and 2017 were retrospectively analysed. A total of 345 patients with missing information on any variable were excluded. Another 14 patients who received adjuvant radiotherapy or adjuvant chemotherapy, 2 patients with other simultaneous malignancies and 1 patient with coexistent hematologic disorders were excluded. A total of 1111 patients were included in the analysis. The flow chart of the inclusion and exclusion process is shown in Fig. 1.

The characteristics of the included patients are listed in Table 1. The median age of the enrolled patients was 56 years. The median observation period was 40 months. During observation, a total of 82 patients died. A total of 1008 (90.7%) patients were diagnosed at stage I or II, while 103 (9.3%) were diagnosed at stage III or IV. The majority of the patients underwent hysterectomy, bilateral salpingo-oophorectomy and lymphadenectomy (78.2%). The residual tumours were present in 34 patients. The major histopathological subtype was endometrioid endometrial carcinoma (91.9%).

**Table 1:** Associations of the NLR, PLR and MLR with other clinicopathological variables

Clinicopathologic Characteristics	Total	Low NLR	High NLR	P	Low PLR	High PLR	P	Low MLR	High MLR	P
	(***n*** = 1111), No (%)	(***n*** = 612, 55.1%), No (%)	(***n*** = 499, 44.9%), No (%)		(***n*** = 647, 58.2%), No (%)	(***n*** = 464, 41.8%), No (%)		(***n*** = 687, 61.8%), No (%)	(***n*** = 424, 38.2%), No (%)	
Age, y										
<55	467 (42.0)	235 (38.4)	232 (46.5)	0.003	223 (34.5)	244 (52.6)	<0.001	276 (40.2)	191 (45.0)	0.019
55–64	488 (43.9)	298 (48.7)	190 (38.1)		326 (50.4)	162 (34.9)		324 (47.2)	164 (38.7)	
65–74	131 (11.8)	69 (11.3)	62 (12.4)		85 (13.1)	46 (9.9)		76 (11.1)	55 (13.0)	
≥ 75	25 (2.3)	10 (1.6)	15 (3.0)		13 (2.0)	12 (2.6)		11 (1.6)	14 (3.3)	
Stage										
I	926 (83.3)	523 (85.5)	403 (80.8)	0.027	562 (86.9)	364 (78.4)	<0.001	592 (86.2)	334 (78.8)	0.003
II	82 (7.4)	43 (7.0)	39 (7.8)		39 (6.0)	43 (9.3)		44 (6.4)	38 (9.0)	
III	86 (7.7)	42 (6.9)	44 (8.8)		43 (6.6)	43 (9.3)		46 (6.7)	40 (9.4)	
IV	17 (1.5)	4 (0.7)	13 (2.6)		3 (0.5)	14 (3.0)		5 (0.7)	12 (2.8)	
Grade										
1	510 (45.9)	285 (46.6)	225 (45.1)	0.19	298 (46.1)	212 (45.7)	<0.001	314 (45.7)	196 (46.2)	0.043
2	378 (34.0)	216 (35.3)	162 (32.5)		246 (38.0)	132 (28.4)		249 (36.2)	129 (30.4)	
3	223 (20.1)	111 (18.1)	112 (22.4)		103 (15.9)	120 (25.9)		124 (18.1)	99 (23.4)	
BMI, kg/m^2^										
<25	467 (42.0)	241 (39.4)	226 (45.3)	0.096	258 (39.9)	209 (45.0)	0.192	284 (41.3)	183 (43.2)	0.406
25–30	547 (49.2)	319 (52.1)	228 (45.7)		333 (51.5)	214 (33.1)		337 (49.1)	210 (49.5)	
≥ 30	97 (8.7)	52 (8.5)	45 (9.0)		56 (8.7)	41 (8.8)		66 (9.6)	31 (7.3)	
Diabetes										
Absent	918 (82.6)	498 (81.4)	420 (84.2)	0.221	526 (81.3)	392 (84.5)	0.167	560 (81.5)	358 (84.4)	0.212
Present	193 (17.4)	114 (18.6)	79 (15.8)		121 (18.7)	72 (15.5)		127 (18.5)	66 (15.6)	
Lymphovascular space invasion										
Absent	1081 (97.3)	597 (97.5)	484 (97.0)	0.57	631 (97.5)	450 (97.0)	0.581	668 (97.2)	413 (97.4)	0.864
Present	30 (2.7)	15 (2.5)	15 (3.0)		16 (2.5)	14 (3.0)		19 (2.8)	11 (2.6)	
Histopathological subtype										
Endometrioid	1021 (91.9)	572 (93.5)	449 (90.0)	0.297	610 (94.3)	411 (88.6)	0.025	1021 (91.9)	384 (90.6)	0.427
Stromal sarcoma	4 (0.4)	1 (0.2)	3 (0.6)		2 (0.3)	2 (0.4)		4 (0.4)	1 (0.2)	
Clear cell	6 (0.5)	2 (0.3)	4 (0.8)		2 (0.3)	4 (0.9)		6 (0.5)	4 (0.9)	
Serous	25 (2.3)	10 (1.6)	15 (3.0)		12 (1.9)	13 (2.8)		25 (2.3)	13 (3.1)	
Mixed	25 (2.3)	12 (2.0)	13 (2.6)		10 (1.5)	15 (3.2)		25 (2.3)	9 (2.1)	
Carcinosarcoma	30 (2.7)	15 (2.5)	15 (3.0)		11 (1.7)	19 (4.1)		30 (2.7)	13 (3.1)	

*NLR* neutrophil*: lymphocyte ratio; PLR* platelet*: lymphocyte ratio; MLR* monocyte: lymphocyte ratio

**Fig. 2:**
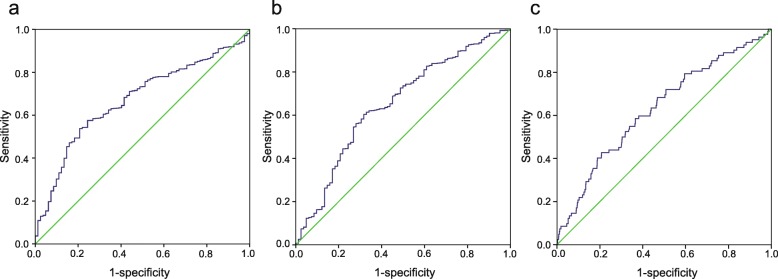
Receiver operating characteristics (ROC) curve analysis of the NLR, PLR and MLR. **a** ROC curve analysis of the NLR for OS in endometrial cancer patients. **b** ROC curve analysis of the PLR for OS in endometrial cancer patients. **c** ROC curve analysis of the MLR for OS in endometrial cancer patients

The median NLR, PLR and MLR were 2.01 (range 0.52–60.44), 121.11 (range 24.06–634.48), and 0.19 (range 0.01–0.83), respectively. ROC curves were generated to identify the optimal cutoffs for the NLR, PLR, and MLR (Fig. 2). A cutoff of 2.14 (area under the curve, AUC = 0.671) for the NLR had the highest Youden index. Similarly, cutoffs of 131.82 (AUC = 0.652) and 0.22 (AUC = 0.630) were identified for the PLR and MLR, respectively. For further analysis, we dichotomized patients into low and high groups by the cutoffs. Regarding the NLR cutoff, 612 patients (55.1%) were in the low group, whereas 499 (44.9%) were in the high group. Regarding the PLR, 647 (58.2%) patients were in the low group, while 464 (41.8%) were in the high group. Regarding the MLR, 687 (61.8%) patients had a low MLR, and 424 (38.2%) had a high MLR. Chi-square tests (Table 1, *P* < 0.05) showed that the NLR was significantly associated with patient age and clinical stage, and the PLR was significantly associated with patient age, clinical stage, tumour grade and histopathological subtype. Moreover, significant corrections of the MLR with patient age, clinical stage and tumour grade were revealed.

**Fig. 3:**
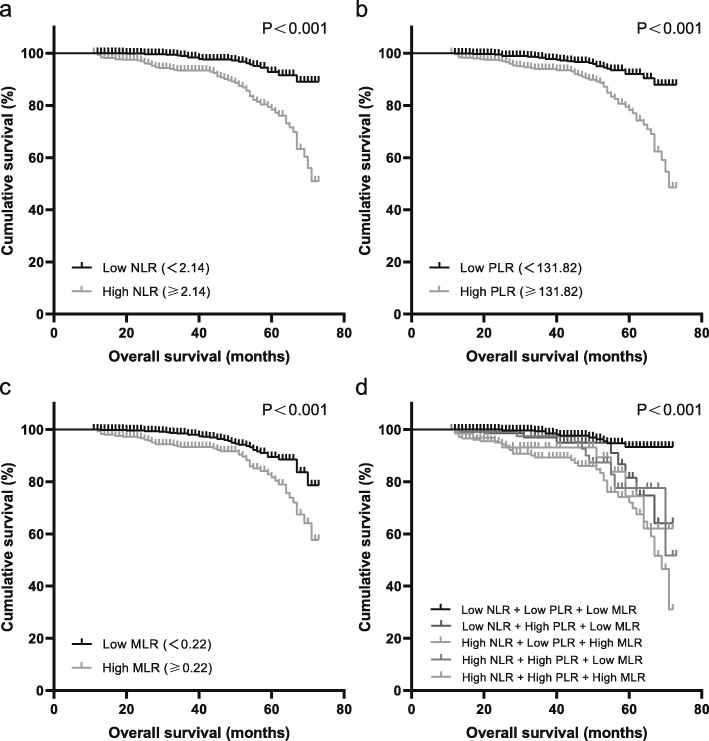
Overall survival of included patients stratified according to the preoperative NLR, PLR and MLR cutoffs. **a** Kaplan-Meier curves and *P* values indicate the relation between OS and the NLR (*P* < 0.001). **b** The relation between OS and the PLR (*P* < 0.001). **c** The relation between OS and the MLR (*P* < 0.001). **d** The OS comparison of the combined indicators (*P* < 0.001)

Table 2 shows that a high NLR, PLR and MLR were associated with poor OS both in the univariate analysis and the multivariate analysis (*P* < 0.05). In the multivariate analysis, the HRs of a high NLR, PLR and MLR were 2.71 (95% CI = 1.83–4.02, P<0.001), 2.75 (95% CI = 1.90–3.97, P<0.001), and 1.72 (95% CI = 1.20–2.45, *P* = 0.003), respectively. By contrast, the combined indicators, high NLR + high PLR + high MLR, high NLR + high PLR + low MLR, low NLR + high PLR + low MLR, and high NLR + low PLR + high MLR, showed good prognostic value in both the univariate and multivariate analyses. Other combinations were insignificant, as shown in the table. Some clinical characteristics, including patients older than 64 years, patients with Stage III to IV or Grade 2 to 3, patients with lymphovascular space invasion and patients with carcinosarcoma or a mixed histopathological subtype, were also significantly associated with OS in both the univariate and multivariate analyses. As the multicollinearity was detected as low level, all the variables were retained in the model. Besides, no violations of proportional hazards or other model assumptions were found by testing Schoenfeld residuals. Kaplan-Meier analysis (Fig. 3) indicated that patients with high preoperative NLR, PLR or MLR had significantly worse OS. The cumulative 5-year OS rate in the high NLR group was significantly lower than that in the low NLR group (77.9% vs 92.1%, Fig. 3a). The 5-year OS rate in the high PLR group was significantly lower than that in the low PLR group (77.2% vs 92.0%, Fig. 3b). The 5-year OS rate in the high MLR group was significantly lower than that in the low MLR group (82.2% vs 88.5%, Fig. 3c). For the combined indicators, the 5-year OS rate in the high NLR + high PLR + high MLR group was the lowest among all groups (72.4%, Fig. 3d). The supporting information in Fig. S1 and Fig. S2 showed the combined indicators had stronger connection with lower OS rates both in early stages (Stage I and II) and advanced stages (Stage III and IV).

**Table 2:** Overall survival of the preoperative NLR, PLR and MLR with other clinicopathological variables

Clinicopathologic Characteristics	Univariate Analysis, HR (95% CI)	P	Multivariate Analysis, HR (95% CI)	P
Age, y				
<55	1.00		1.00	
55–64	0.91 (0.62–1.34)	0.622	0.99 (0.66–1.49)	0.969
65–74	2.27 (1.40–3.67)	0.001	2.57 (1.56–4.23)	<0.001
≥ 75	3.69 (1.92–7.11)	<0.001	2.86 (1.44–5.68)	0.003
Stage				
I	1.00		1.00	
II	2.02 (1.17–3.49)	0.012	1.34 (0.76–2.37)	0.314
III	6.89 (4.69–10.12)	<0.001	3.26 (2.13–4.98)	<0.001
IV	11.29 (6.33–20.14)	<0.001	2.68 (1.41–5.10)	0.003
Grade				
1	1.00		1.00	
2	2.43 (1.43–4.12)	0.001	1.85 (1.06–3.23)	0.030
3	8.54 (5.21–13.99)	<0.001	3.80 (2.22–6.51)	<0.001
BMI, kg/m^2^				
<25	1.00		1.00	
25–30	1.21 (0.80–1.82)	0.367	1.19 (0.78–1.82)	0.409
≥ 30	0.89 (0.48–1.66)	0.713	1.07 (0.57–2.03)	0.828
Diabetes				
Absent	1.00		1.00	
Present	1.04 (0.65–1.68)	0.854	0.94 (0.57–1.54)	0.794
Lymphovascular space invasion				
Absent	1.00		1.00	
Present	6.75 (4.19–10.88)	<0.001	3.11 (1.86–5.20)	<0.001
Histopathological subtype				
Endometrioid	1.00		1.00	
Stromal sarcoma	2.34 (0.32–16.80)	0.399	0.34 (0.04–2.83)	0.321
Clear cell	2.69 (0.37–19.35)	0.325	0.71 (0.09–5.26)	0.733
Serous	6.91 (4.10–11.63)	<0.001	1.02 (0.55–1.89)	0.939
Mixed	4.87 (2.45–9.69)	<0.001	2.76 (1.31–5.79)	0.007
Carcinosarcoma	7.76 (4.72–12.75)	<0.001	2.80 (1.61–4.87)	<0.001
NLR				
<2.14	1.00		1.00	
≥ 2.14	3.96 (2.72–5.78)	<0.001	2.71 (1.83–4.02)	<0.001
PLR				
<131.82	1.00		1.00	
≥ 131.82	3.54 (2.47–5.08)	<0.001	2.75 (1.90–3.97)	<0.001
MLR				
<0.22	1.00		1.00	
≥ 0.22	2.12 (1.52–2.97)	<0.001	1.72 (1.20–2.45)	0.003
Combined NLR + PLR + MLR				
NLR low + PLR low + MLR low	1.00		1.00	
NLR low + PLR high + MLR high	1.02 (1.57–6.59E157)	0.952	<0.001 (0.000–5.56E197)	0.961
NLR low + PLR low + MLR high	0.47 (0.10–1.83)	0.252	0.40 (0.09–1.74)	0.225
NLR high + PLR low + MLR low	2.07 (0.96–4.45)	0.063	1.42 (0.64–3.15)	0.391
NLR high + PLR low + MLR high	3.83 (1.86–7.89)	<0.001	2.41 (1.13–5.15)	0.023
NLR low + PLR high + MLR low	3.92 (2.01–7.62)	<0.001	2.59 (1.31–5.11)	0.006
NLR high + PLR high + MLR low	3.80 (1.93–7.47)	<0.001	2.96 (1.50–5.89)	0.002
NLR high + PLR high + MLR high	7.04 (4.22–11.76)	<0.001	4.34 (2.54–7.42)	<0.001

*CI, confidence interval; HR, hazard ratio; NLR, neutrophil: lymphocyte ratio; PLR, platelet: lymphocyte ratio; MLR, monocyte: lymphocyte*

In the subgroup analysis (full results are provided in supporting information Table S1 and Table S2) of the early (I/II) and advanced (III/IV) stages, the NLR, PLR, and MLR were significantly associated with worse OS in both the early and advanced stages in the univariate analysis. In the multivariate analysis, after adjusting for age, stage, grade, BMI, diabetes, histopathological subtype and lymphovascular space invasion, the NLR, PLR, and MLR were independently prognostic in the early stage (*P* < 0.05), and the PLR and MLR were independently prognostic in the advanced stage (P < 0.05). By contrast, the prognostic values of the combined indicators were consistent in all stages both in the univariate and multivariate analyses. The combined high NLR + high PLR high + high MLR group was associated with the worst OS among all groups (for the multivariate analysis, in early stage, HR = 4.26, 95% CI = 2.25–8.08, P<0.001; in advanced stage, HR = 8.91, 95% CI = 2.97–26.72, P<0.001).

**Fig. 4:**
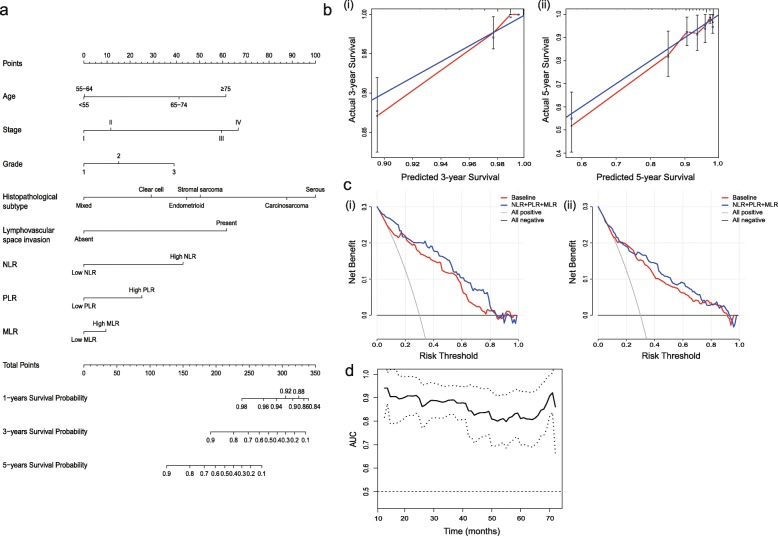
Nomogram of the prognostic model. **a** Nomogram of the prognostic model. **b** Calibration curve evaluating the calibration of nomogram according to the agreement between the predicted OS and observed outcome. **i** The predicted 3-year OS and observed outcome. **ii** The predicted 5-year OS and observed outcome. *The y-axis represents actual survival estimated by the nomogram, and the x-axis represents the predicted probability of survival. The reference line represents a perfect prediction by an ideal model.***c i** The DCA curves of the nomograms compared for 3-year OS. **ii** The DCA curves of the nomograms compared for 5-year OS. *The all negative line indicates that no patients survive. The all positive line indicates that all patients survive. The NLR + PLR + MLR line shows the clinical net benefits of all factors, including age, stage, grade, histopathological subtype, lymphovascular space invasion, NLR, PLR, and MLR, while the baseline shows the clinical net benefits of all factors excluding NLR, PLR, and MLR.***d** The AUC of time-dependent ROC of the nomogram

To evaluate the prognostic value of the combined indicators (NLR, PLR and MLR) in EC, a nomogram analysis of OS was performed by the multivariate Cox regression model (Fig. 4a). By summing the points assigned to each variable and drawing a straight line from the total point axis, 1-year probability of survival, 3-year probability of survival and 5-year probability of survival were predicted. The calibration curves showed predicted probability versus actual probability of 3-year and 5-year OS (Fig. 4b-i, ii). The resullt showed an optimal agreement between the prediction using nomogram and the actual observed survival. Furthermore, the Harrell’s C-index, the decision curve analysis (DCA), and the AUC of time-dependent ROC curves were used to evaluate the efficacy of the predictive model. The Harrell’s C-index of the nomogram was 0.847 (95% CI = 0.804–0.890), but the C-index of the model without the involvement of NLR, PLR and MLR declined to 0.803 (95% CI = 0.744–0.862). DCA was performed to calculate the clinical net benefit of each model and it was found that the model with the involvement of NLR, PLR and MLR was more benefit than that of the model without NLR, PLR, and MLR in predicting 3-year and 5-year OS (Fig. 4c-i, ii). The AUC of time-dependent ROC (Fig. 4d) remained in a high place and showed that the nomogram was of good efficacy for predicting prognosis of EC patients with different survival time.

## Discussion

In our study, elevated NLR, PLR and MLR were independent prognostic factors for EC. The findings are consistent with some previous studies [15, 16] discussing the prognostic value of the NLR, PLR and MLR for EC, but none of them focused on the combination of the above ratios. Our further combined studies showed that the low NLR + high PLR + high MLR group, the low NLR + low PLR + high MLR group, and the high NLR + low PLR + low MLR group were not significantly associated with OS, indicating that a mere increase in the NLR or PLR or MLR without significant changes in the other two indicators would not provide a convincing prognostic value. By contrast, the HRs of a high NLR + a high PLR + a high MLR showed greater prognostic significance and less change in the univariate and multivariate analyses than a single ratio, indicating a more stable prognostic value against other influencing factors.

The present study is, to the best of our knowledge, the first one to discuss the role of the combined preoperative ratios, NLR, PLR, and MLR, in evaluating the prognosis of EC patients. Some previous studies [11, 12] had revealed the lack of reliability of the prognostic value of a single ratio in EC. In other cancer models, some scholars [17, 18] also doubted the prognostic value of a single ratio and recommended combined ratios as better prognostic indicators. Therefore, it is of great significance to combine the NLR, PLR, and MLR in the evaluation of prognosis to raise diagnostic accuracy.

Besides the three ratios, we also found that some clinical characteristics, including patients older than 64 years, patients with Stage III to IV or Grade 2 to 3, patients with LVSI and patients with carcinosarcoma or a mixed histopathological subtype, were also significantly associated with OS in both the univariate and multivariate analyses. The findings matched the existing studies. Yen’s study [19] showed a worse outcome in patients with older age, high-risk histology or high-grade EC after primary surgery. Bendifallah [20] incorporated LVSI into the ESMO classification and achieved better defined indications for EC patients. By contrast, BMI and diabetes did not show a significant association with prognosis in our study. In Mauland’s study [21], high BMI is significantly associated with non-aggressive disease in endometrial cancer in the univariate analysis, but not in the multivariate analysis. Binder [22] indicated endometrial cancer was associated with medical comorbidities such as obesity and diabetes, but Mitsuhashi [23] suggested the use of metformin, an antidiabetic drug, could inhibit endometrial cancer cell growth. Therefore the medication usage of EC patients with comorbidities might affect their prognosis and warrant further study. To make the nomogram model more comprehensive, we involved both BMI and diabetes in the assessment system.

The establishment of a nomogram model makes it visible and qualified to evaluate the prognosis with NLR, PLR, MLR and other significant indicators during clinical practice. The model has the advantage of integrating diverse relevant determinants into the prognosis and estimating the individualized risk based on the characteristics of each patient, which usually works better than the subjective judgment of a clinician [24]. In our study, we generated a nomogram model consisting of age, clinical stage, tumour grade, histopathological subtype, lymphovascular space invasion, NLR, PLR and MLR, which could scientifically prognosticate the survival of EC patients by combining blood markers and clinicopathological factors. The calibration curves of 3-year probability of survival and 5-year probability of survival showed that the nomogram had good discrimination and calibration. Ouldamer [25] had reported a nomogram predicting poor prognosis recurrence (PPR) in EC patients, but she did not incorporate NLR, PLR and MLR, and the Harrell’s C-index was 0.82 (95% CI = 0.73–0.89). By contrast, the Harrell’s C-index was 0.847 (95% CI = 0.804–0.890) in our study, which is also better than similar studies [26, 27] focusing on the relationship between cancers and those three ratios. Figure 4a seemed to show that MLR contributed less to the total Nomogram score, but the Harrell’s C-index of the nomogram without MLR declined to 0.846 (95% CI = 0.803–0.889), indicating that the addition of MLR enabled more accurate prognosis prediction. The AUC of time-dependent ROC of the nomogram is also taken to evaluate the efficacy of the nomogram in some studies [28]. The AUC of our model was 0.794 but dropped to 0.777 if MLR was excluded.

Currently, the underlying mechanisms of the associations between the three markers and EC remain poorly understood. Some scholars attribute the association to tumour-induced inflammation and host immunoreaction. Tumour cells could increase peripheral neutrophil levels via stimulating the release of granulocyte colony-stimulating factor (GCSF). In turn, neutrophils promote tumour invasion and metastasis via the release of several growth factors such as vascular endothelial growth factor (VEGF) and proteases such as elastases [29]. On the other hand, neutrophils suppress tumour progression via the debridement of hypoxic tumour cells [30]. The hypercoagulable state significantly accounts for a high mortality in cancer patients. Meanwhile, activated platelets release a mass of molecules, such as TXA2, PGE2, α-granule contents and exosomes, forming a complex tumour microenvironment that supports the progression of tumour cells. Platelets also aggregate surrounding tumour cells and protect them from elimination by immune cells [31]. Monocytes usually migrate into the tumour microenvironment via VEGF and then differentiate into tumour-associated macrophages (TAMs) induced by tumour chemotactic effects. TAMs produce various factors, such as tumour growth factors and angiogenic factors, to accelerate tumour progression and invasion. On the other hand, monocytes can interfere with the proliferation and activation of lymphocytes, leading to the immune suppression of tumour cells [32]. By contrast, the lymphocyte is usually known as an antitumor effector. In general, CD8 + T lymphocytes play a vital role in attacking tumour cells via cytotoxicity. A lack of CD3 + or CD8 + T lymphocytes in EC was usually believed to be related to poor prognosis [33]. In light of the uncertain mechanisms and some of the bidirectional indicators above, the combination of NLR, PLR and MLR may be a superior prognostic factor of EC that reflects the actual status of tumour-associated immunoreaction.

There are some limitations to our study. First, it was a retrospective study conducted in a single institution, although it is by far the largest study (*n* = 1111) to evaluate the prognostic value of preoperative NLR, PLR and MLR. Secondly, all data on preoperative indicators were collected from patients who later underwent surgical treatment. Therefore, the results might not represent the prognostic value for EC patients with unresectable tumours or other interventions.

## Conclusions

In summary, the findings of our study indicate that the preoperative blood NLR, PLR, and MLR are independent prognostic markers for OS in EC patients. The combination of NLR, PLR, and MLR provides better prognostic value than any single ratio. The nomogram involving the combination of NLR, PLR, MLR and other clinicopathological factors is recommended as a more convenient and practical model to predict OS for EC patients clinically.

## Supplementary information

**Additional file 1 Table S1** Univariate and multivariate analysis of the ratios with other clinicopathological variables in early stages

**Additional file 2 Table S2** Univariate and multivariate analysis of the ratios with other clinicopathological variables in advanced stages

**Additional file 3 Figure S1** Overall survival of patients in early stages stratified according to preoperative NLR, PLR, MLR cut-offs. **a** Kaplan-Meier curves and log-rank *P*-values indicated the relation between OS and NLR (*P* < 0.001). **b** The relation between OS and PLR (*P* < 0.001). **c** The relation between OS and MLR (*P* < 0.001). **d** The OS comparison of combined indicators (*P* < 0.001).

**Additional file 4 Figure S2** Overall survival of patients in advanced stage stratified according to preoperative NLR, PLR, and MLR cut-offs. **a** Kaplan-Meier curves and log-rank P-values indicated the relation between OS and NLR (*P* < 0.01). **b** The relation between OS and PLR (*P* = 0.091). **c** The relation between OS and MLR (*P* < 0.05). **d** The OS comparison of combined indicators (*P* < 0.05).

## Data Availability

The data that support the findings of this study are available from the corresponding author upon reasonable request.

## Abbreviations

NLR: Neutrophil-lymphocyte ratio
PLR: Platelet-lymphocyte ratio
MLR: Monocyte-lymphocyte ratio
EC: Endometrial cancer
ROC: Receiver operating characteristic curve
OS: Overall survival
CA-125: Cancer antigen 125
FBC: Full blood count
BMI: Body mass index
LVSI: Lymphovascular space invasion
AUC: Area under the curve
HR: Hazard ratio
CI: Confidence interval
DCA: Decision curve analysis
PPR: Poor prognosis recurrence
GCSF: Granulocyte colony-stimulating factor
VEGF: Vascular endothelial growth factor
TAMs: Tumour-associated macrophages
