# Supplementary material for: Combination of preoperative neutrophil-lymphocyte ratio, platelet-lymphocyte ratio and monocyte-lymphocyte ratio: a superior prognostic factor of endometrial cancer
Source: BMC Cancer. 2020 May 24;20:464. doi: 10.1186/s12885-020-06953-8 (PMC7245911; doi:10.1186/s12885-020-06953-8)
Supplement: Supplementary file 1 — Additional file 1 Table S1 Univariate and multivariate analysis of the ratios with other clinicopathological variables in early stages [file 12885_2020_6953_MOESM1_ESM.doc]

Table S1. Univariate and multivariate analysis of the ratios with other clinicopathological variables in early stages

| **Clinicopathologic Characteristics** | **Univariate Analysis, HR (95% CI)** | **P** | **Multivariate Analysis, HR (95% CI)** | **P** |
| --- | --- | --- | --- | --- |
| Age, y | | | | |
| ＜55 | 1.00 |  | 1.00 |  |
| 55-64 | 0.75 (0.45-1.26) | 0.271 | 0.86 (0.50-1.48) | 0.574 |
| 65-74 | 2.15 (1.16-3.99) | 0.015 | 1.97 (1.05-3.71) | 0.035 |
| ≥75 | 4.90 (2.33-10.29) | ＜0.001 | 2.87 (1.27-6.49) | 0.011 |
| Stage | | | | |
| I | 1.00 |  | 1.00 |  |
| II | 2.02 (1.17-3.48) | 0.012 | 1.40 (0.78-2.51) | 0.253 |
| Grade | | | | |
| 1 | 1.00 |  | 1.00 |  |
| 2 | 1.98 (1.05-3.77) | 0.036 | 2.22 (1.15-4.30) | 0.018 |
| 3 | 8.30 (4.60-14.97) | ＜0.001 | 4.66 (2.48-8.74) | ＜0.001 |
| BMI, kg/m^2^ | | | | |
| ＜25 | 1.00 |  | 1.00 |  |
| 25-30 | 1.07 (0.63-1.84) | 0.796 | 0.97 (0.56-1.68) | 0.909 |
| ≥30 | 1.11 (0.53-2.31) | 0.782 | 1.06 (0.50-2.23) | 0.887 |
| Diabetes | | | | |
| Absent | 1.00 |  | 1.00 |  |
| Present | 1.42 (0.71-2.85) | 0.322 | 0.98 (0.48-2.00) | 0.958 |
| Lymphovascular space invasion | | | | |
| Absent | 1.00 |  | 1.00 |  |
| Present | 9.16 (3.94-21.33) | ＜0.001 | 3.56 (1.48-8.71) | 0.005 |
| Histopathological subtype | | | | |
| Endometrioid | 1.00 |  | 1.00 |  |
| Stromal sarcoma | ＜0.001 (＜0.001-9.00E217) | 0.970 | ＜0.001 (＜0.001-2.58E252) | 0.973 |
| Clear cell | 5.67 (0.78-41.10) | 0.086 | 3.22 (0.41-25.30) | 0.266 |
| Serous | 2.61 (0.64-10.72) | 0.182 | 1.10 (0.26-4.71) | 0.901 |
| Mixed | 4.89 (1.95-12.24) | 0.001 | 3.57 (1.34-9.49) | 0.011 |
| Carcinosarcoma | 9.14 (5.17-16.14) | 0.001 | 2.81 (1.37-5.80) | 0.005 |
| NLR | | | | |
| ＜2.14 | 1.00 |  | 1.00 |  |
| ≥2.14 | 3.61 (2.24-5.81) | ＜0.001 | 2.84(1.75-4.62) | ＜0.001 |
| PLR | | | | |
| ＜131.82 | 1.00 |  | 1.00 |  |
| ≥131.82 | 3.48 (2.19-5.52) | ＜0.001 | 2.84 (1.75-4.62) | ＜0.001 |
| MLR | | | | |
| ＜0.22 | 1.00 |  | 1.00 |  |
| ≥0.22 | 1.75 (1.13-2.70) | 0.012 | 1.68 (1.08-2.61) | 0.022 |
| Combined NLR+PLR+MLR | | | | |
| NLR low + PLR low + MLR low | 1.00 |  | 1.00 |  |
| NLR high + PLR low + MLR low | 2.34 (0.98-5.59) | 0.055 | 1.62 (0.67-3.96) | 0.287 |
| NLR high + PLR low + MLR high | ＜0.001 (＜0.001-1.28E204) | 0.962 | ＜0.001 (＜0.001-1.54E242) | 0.968 |
| NLR high + PLR high + MLR low | 2.96 (1.24-7.06) | 0.015 | 2.11 (0.87-5.15) | 0.100 |
| NLR low + PLR high + MLR low | 2.26 (0.81-6.28) | 0.118 | 1.97 (0.70-5.50) | 0.198 |
| NLR high + PLR high + MLR high | 5.97 (3.22-11.08) | ＜0.001 | 4.26 (2.25-8.08) | ＜0.001 |
| Other combinations | 1.70 (0.76-3.84) | 0.199 | 1.20 (0.52-2.78) | 0.669 |
| y, years; CI, confidence interval; HR, hazard ratio; BMI, kg/m^2^, body mass index; NLR, neutrophil: lymphocyte ratio; PLR, platelet: lymphocyte ratio; MLR, monocyte: lymphocyte ratio; Other combinations, NLR low + PLR low + MLR high or NLR low + PLR high + MLR high. | | | | |
