# Supplementary material for: Combination of preoperative neutrophil-lymphocyte ratio, platelet-lymphocyte ratio and monocyte-lymphocyte ratio: a superior prognostic factor of endometrial cancer
Source: BMC Cancer. 2020 May 24;20:464. doi: 10.1186/s12885-020-06953-8 (PMC7245911; doi:10.1186/s12885-020-06953-8)
Supplement: Supplementary file 2 — Additional file 2 Table S2 Univariate and multivariate analysis of the ratios with other clinicopathological variables in advanced stages [file 12885_2020_6953_MOESM2_ESM.doc]

Table S2. Univariate and multivariate analysis of the ratios with other clinicopathological variables in advanced stages

| **Clinicopathologic Characteristics** | **Univariate Analysis, HR (95% CI)** | **P** | **Multivariate Analysis, HR (95% CI)** | **P** |
| --- | --- | --- | --- | --- |
| Age, y |  |  |  |  |
| ＜55 | 1.00 |  | 1.00 |  |
| 55-64 | 0.98 (0.54-1.79) | 0.954 | 1.19 (0.61-2.30) | 0.612 |
| 65-74 | 2.37 (1.09-5.14) | 0.029 | 3.73 (1.31-10.60) | 0.013 |
| ≥75 | 1.18 (0.27-5.11) | 0.823 | 1.07 (0.19-6.12) | 0.941 |
| Stage |  |  |  |  |
| III | 1.00 |  | 1.00 |  |
| IV | 1.60 (0.87-2.93) | 0.131 | 0.72 (0.35-1.52) | 0.392 |
| Grade |  |  |  |  |
| 1 | 1.00 |  | 1.00 |  |
| 2 | 1.9 (0.43-3.29) | 0.743 | 0.83 (0.26-2.66) | 0.749 |
| 3 | 1.68 (0.63-4.49) | 0.300 | 1.17 (0.40-3.42) | 0.780 |
| BMI, kg/m^2^ | | | | |
| ＜25 | 1.00 |  | 1.00 |  |
| 25-30 | 1.79 (0.92-3.49) | 0.087 | 2.37 (1.13-4.97) | 0.023 |
| ≥30 | 0.75 (0.21-2.69) | 0.654 | 1.70 (0.41-6.97) | 0.463 |
| Diabetes | | | | |
| Absent | 1.00 |  | 1.00 |  |
| Present | 1.01 (0.52-1.96) | 0.984 | 0.67 (0.31-1.44) | 0.302 |
| Lymphovascular space invasion | | | | |
| Absent | 1.00 |  | 1.00 |  |
| Present | 1.51 (0.82-2.78) | 0.189 | 2.08 (1.05-4.09) | 0.035 |
| Histopathological subtype | | | | |
| Endometrioid | 1.00 |  | 1.00 |  |
| Stromal sarcoma | 1.92 (0.26-14.24) | 0.522 | 0.62 (0.04-8.90) | 0.724 |
| Clear cell | ＜0.001 (1.23E-289-2.18E279) | 0.974 | ＜0.001 (1.22E-311-1.98E301) | 0.975 |
| Serous | 2.36 (1.26-4.43) | 0.007 | 1.34 (0.53-3.36) | 0.535 |
| Mixed | 2.16 (0.75-6.18) | 0.152 | 1.28 (0.36-4.61) | 0.705 |
| Carcinosarcoma | 4.58 (1.58-13.27) | 0.005 | 2.82 (0.70-11.40) | 0.145 |
| NLR | | | | |
| ＜2.14 | 1.00 |  | 1.00 |  |
| ≥2.14 | 3.41 (1.83-6.35) | ＜0.001 | 1.89 (0.88-4.10) | 0.105 |
| PLR | | | | |
| ＜131.82 | 1.00 |  | 1.00 |  |
| ≥131.82 | 2.38 (1.33-4.25) | 0.003 | 2.21 (1.06-4.59) | 0.034 |
| MLR | | | | |
| ＜0.22 | 1.00 |  | 1.00 |  |
| ≥0.22 | 2.44 (1.41-4.22) | 0.001 | 2.38 (1.16-4.91) | 0.018 |
| Combined NLR+PLR+LMR | | | | |
| NLR low + PLR low + LMR high | 1.00 |  | 1.00 |  |
| NLR low + PLR high + LMR low | 2.31 (0.70-7.63) | 0.171 | 2.23 (0.63-7.96) | 0.215 |
| NLR high + PLR low + MLR low | 1.09 (0.21-5.62) | 0.922 | 0.81 (0.4-4.82) | 0.818 |
| NLR high + PLR high + MLR low | 3.00 (0.94-9.54) | 0.062 | 2.63 (0.78-8.89) | 0.120 |
| NLR high + PLR low + MLR high | 5.08 (1.59-16.25) | 0.006 | 5.02 (1.23-20.45) | 0.024 |
| NLR high + PLR high + MLR high | 5.91 (2.28-15.42) | ＜0.001 | 8.91 (2.97-26.72) | ＜0.001 |
| Other combinations | 0.69 (0.13-3.59) | 0.660 | 0.95 (0.17-5.38) | 0.957 |
| y, years; CI, confidence interval; HR, hazard ratio; BMI, kg/m^2^, body mass index; NLR, neutrophil: lymphocyte ratio; PLR, platelet: lymphocyte ratio; MLR, monocyte: lymphocyte ratio; Others combinations, NLR low + PLR low + MLR high or NLR low + PLR high + MLR high. | | | | |
