# Supplementary figures and images for: Combination of preoperative neutrophil-lymphocyte ratio, platelet-lymphocyte ratio and monocyte-lymphocyte ratio: a superior prognostic factor of endometrial cancer
Source: BMC Cancer. 2020 May 24;20:464. doi: 10.1186/s12885-020-06953-8 (PMC7245911; doi:10.1186/s12885-020-06953-8)

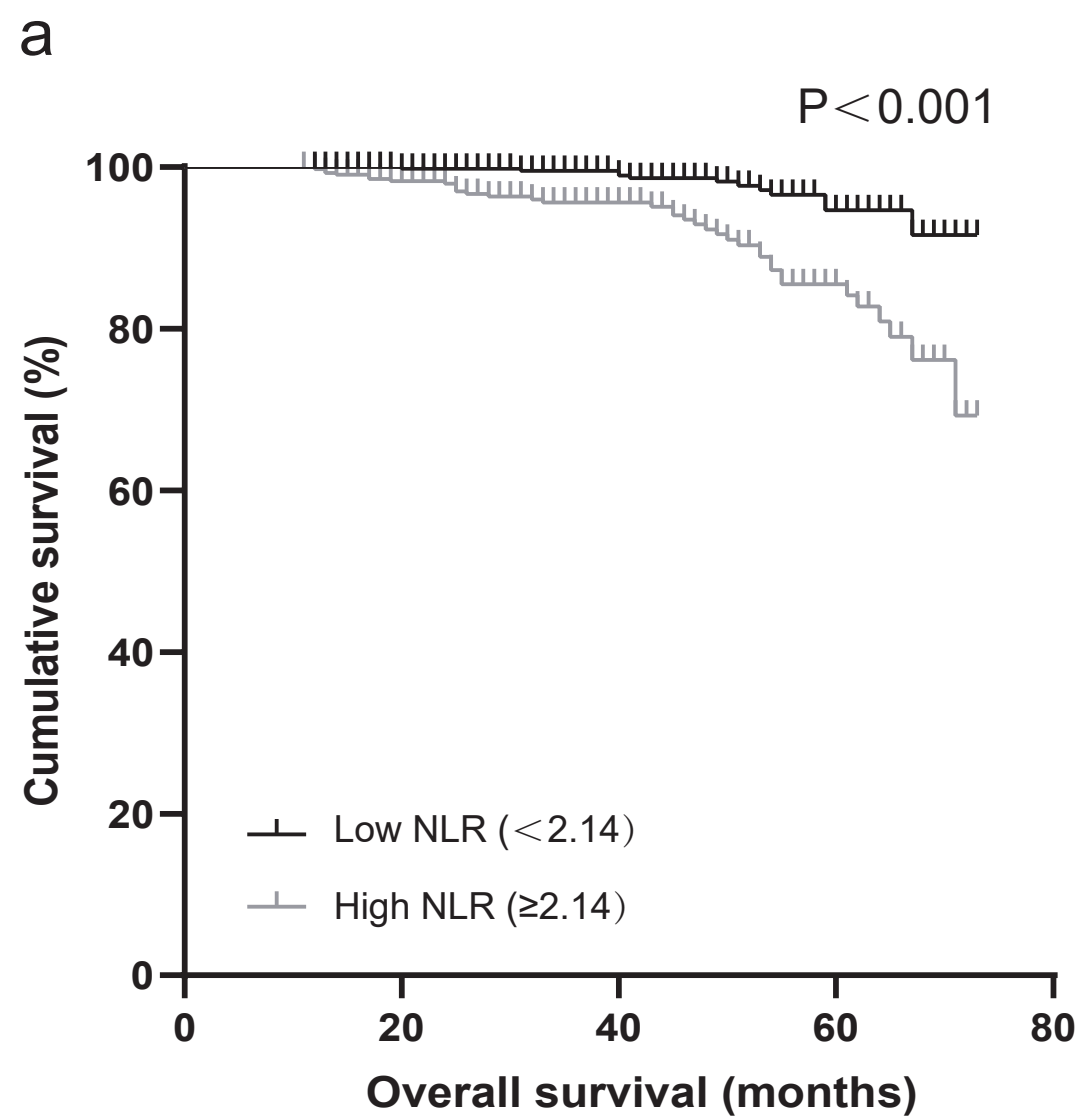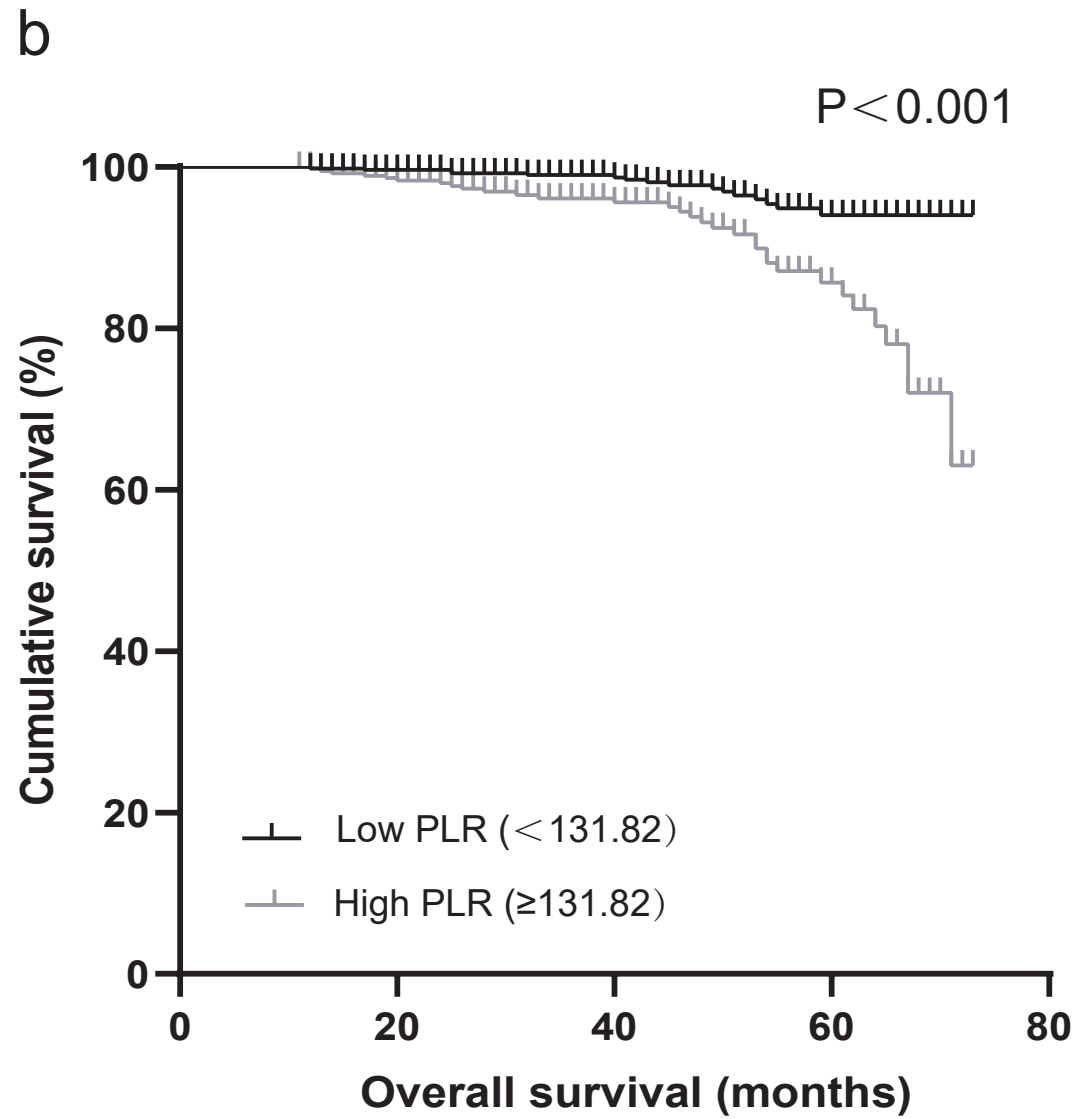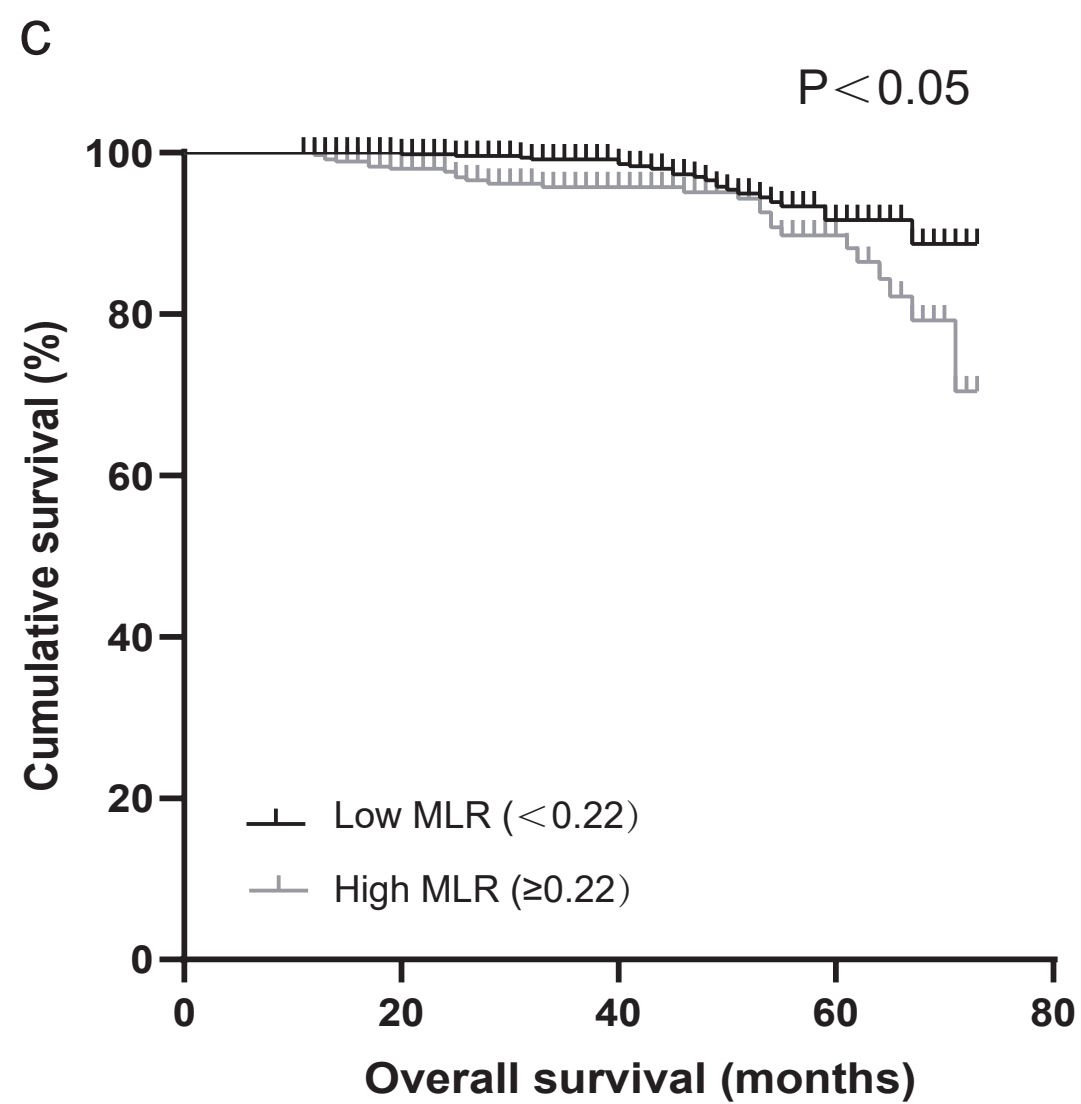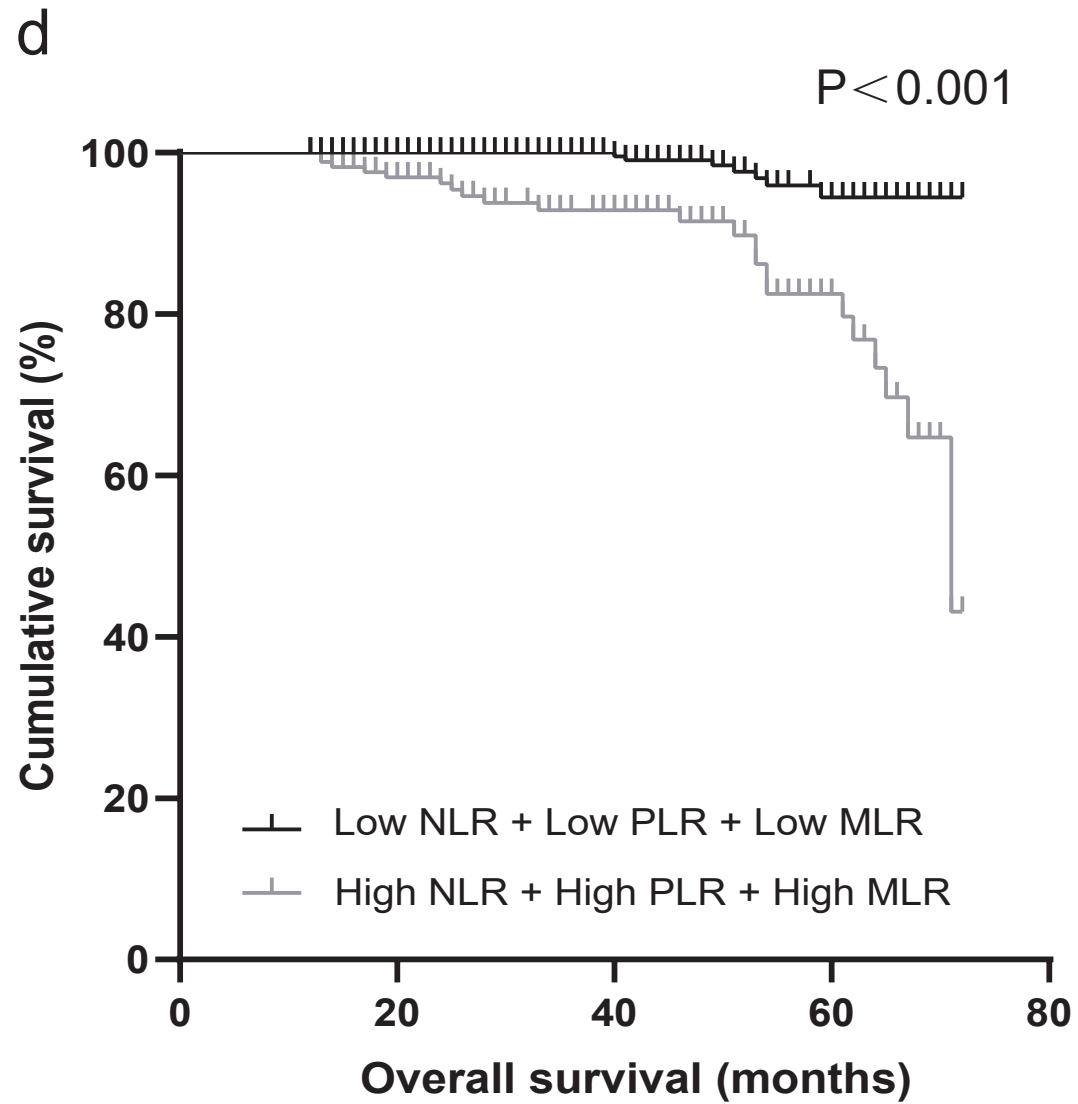

Supplement: Supplementary file 3 — Additional file 3 Figure S1 Overall survival of patients in early stages stratified according to preoperative NLR, PLR, MLR cut-offs. a Kaplan-Meier curves and log-rank P-values indicated the relation between OS and NLR (P < 0.001). b The relation between OS and PLR (P < 0.001). c The relation between OS and MLR (P < 0.001). d The OS comparison of combined indicators (P < 0.001). [file 12885_2020_6953_MOESM3_ESM.pdf]

a

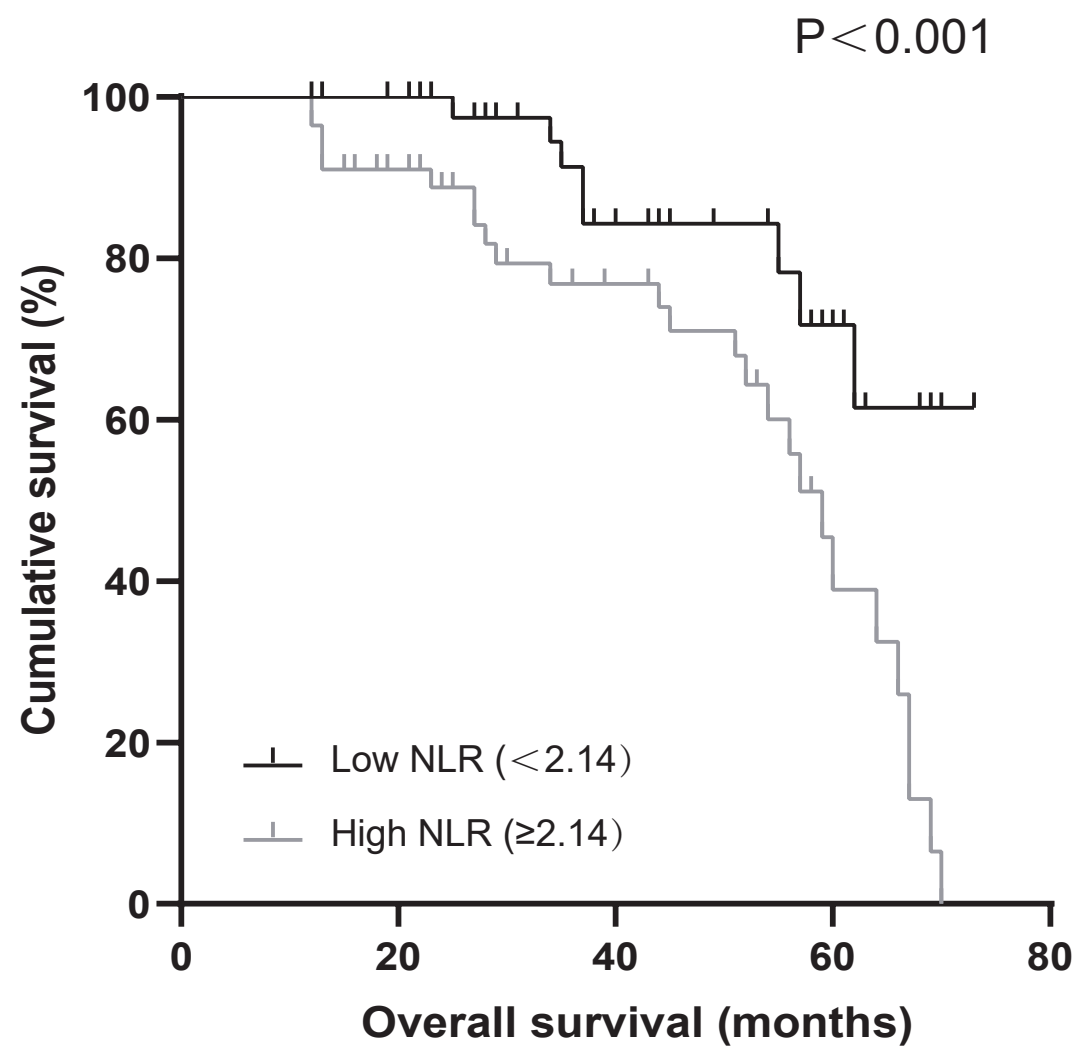

b

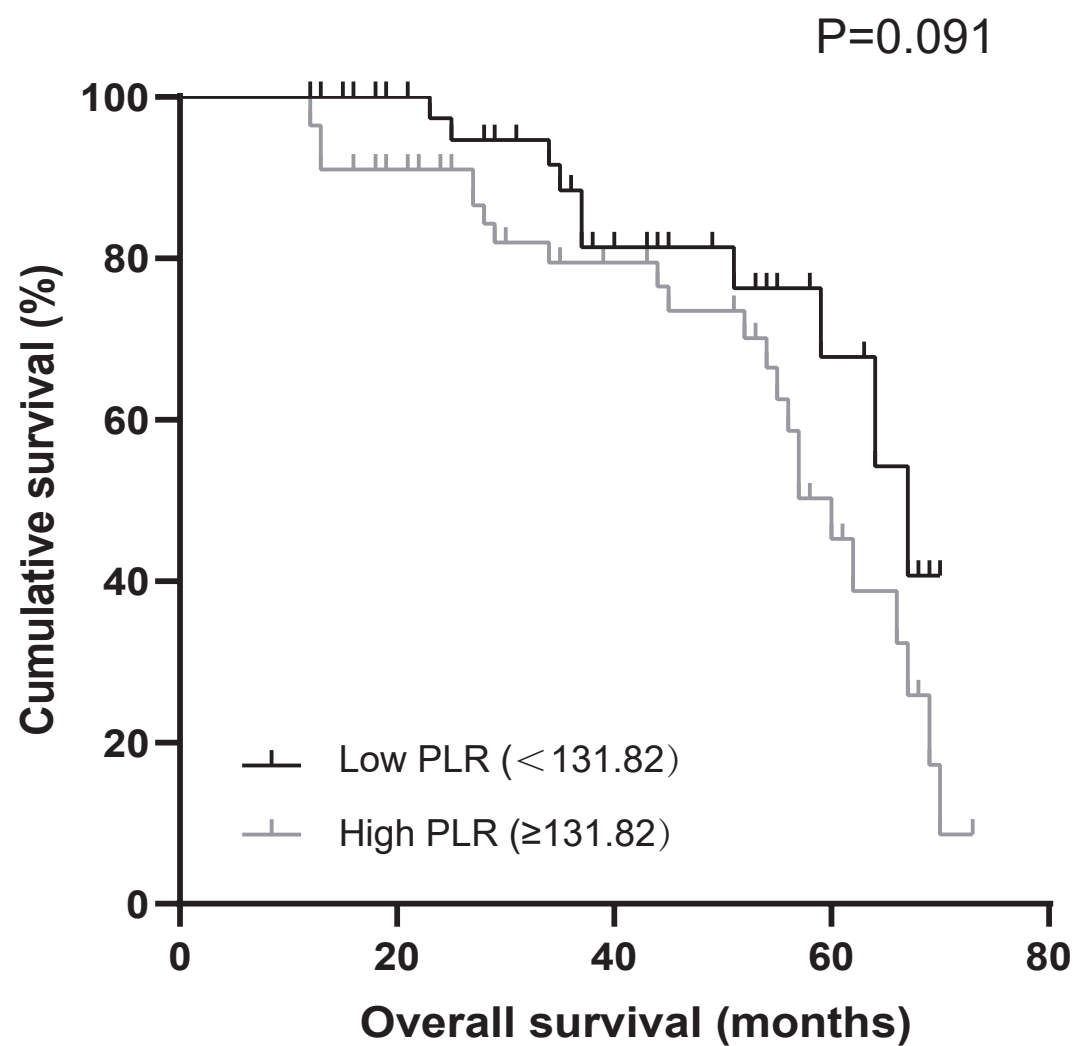

c

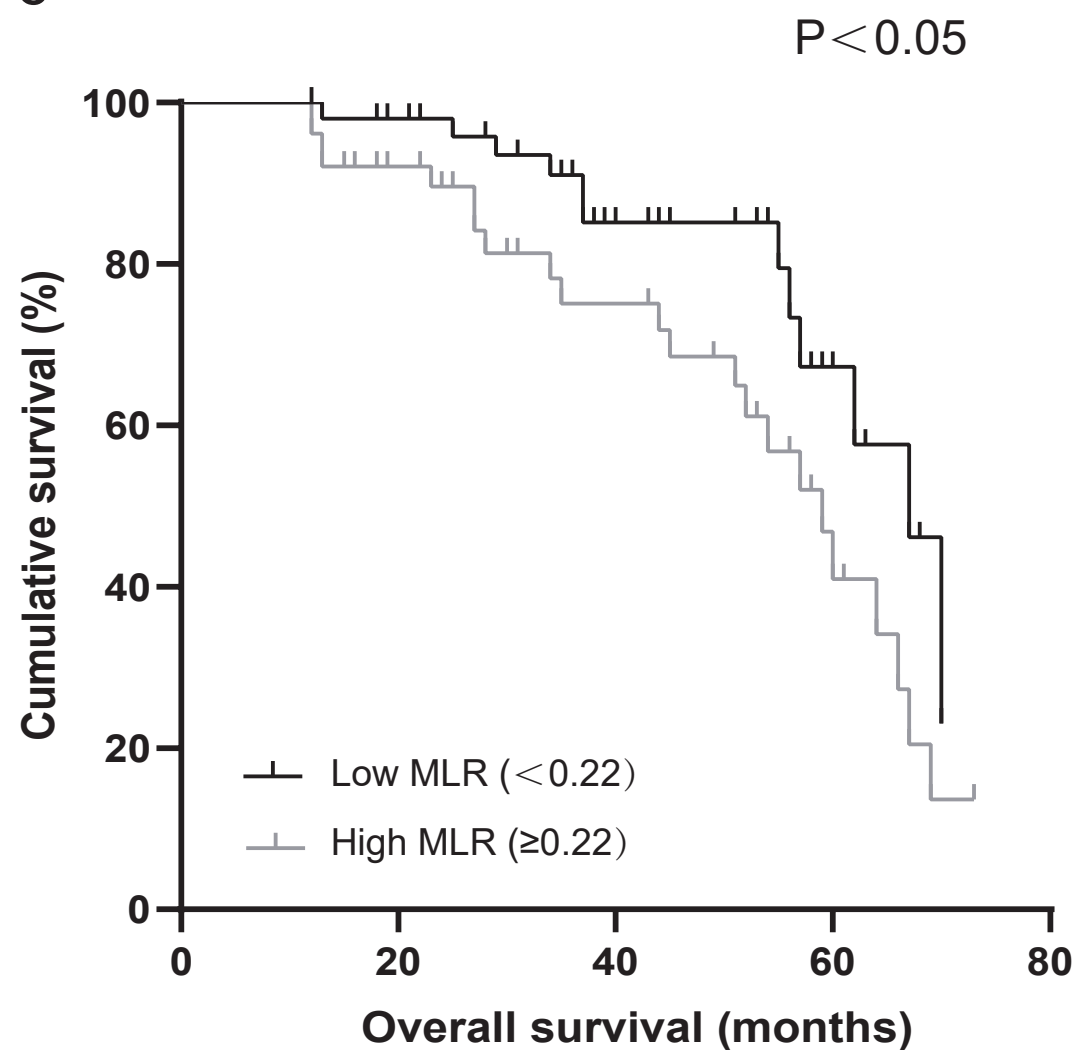

d

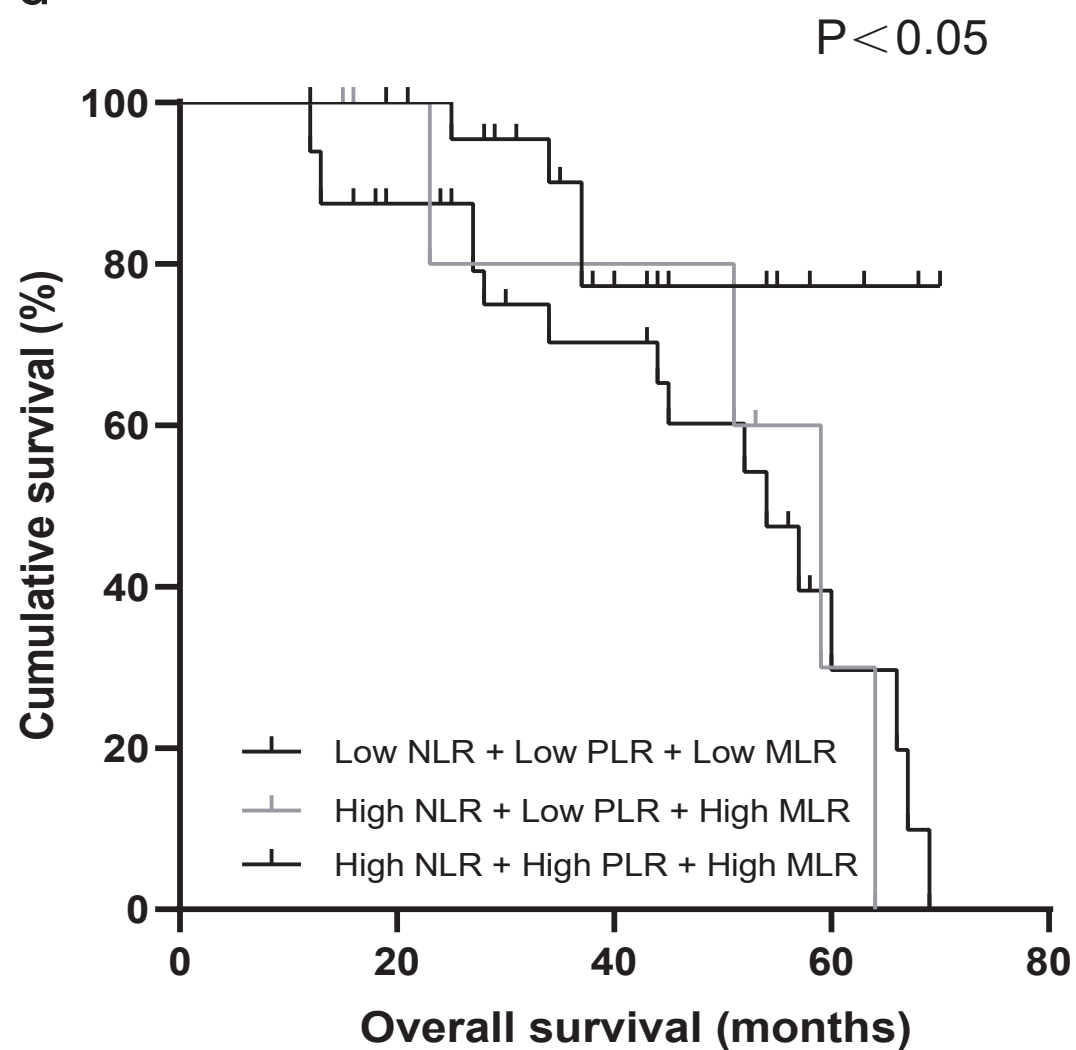

Supplement: Supplementary file 4 — Additional file 4 Figure S2 Overall survival of patients in advanced stage stratified according to preoperative NLR, PLR, and MLR cut-offs. a Kaplan-Meier curves and log-rank P-values indicated the relation between OS and NLR (P < 0.01). b The relation between OS and PLR (P = 0.091). c The relation between OS and MLR (P < 0.05). d The OS comparison of combined indicators (P < 0.05). [file 12885_2020_6953_MOESM4_ESM.pdf]
